# Safety assessment of glutaminase from *Aspergillus niger*

**DOI:** 10.1002/fsn3.1426

**Published:** 2020-02-18

**Authors:** Trung Duc Vo, Christina Sulaiman, Shahrzad Tafazoli, Barry Lynch, Ashley Roberts, Go Chikamatsu

**Affiliations:** ^1^ Intertek Health Sciences Inc. Mississauga ON Canada; ^2^ AR Toxicology Inc. Oakville ON Canada; ^3^ Shin Nihon Chemical Co., Ltd. Anjo Japan

**Keywords:** genotoxicity, glutaminase, subchronic, toxicology

## Abstract

Glutaminase (glutamine aminohydrolase EC 3.5.1.2) is used in the production of food ingredients rich in l‐glutamic acid that are added to finished foods for the purpose of enhancing or improving the savory flavor profile of food. The glutaminase enzyme preparation evaluated in these studies, designated as Sumizyme GT hereafter, is obtained by fermentation of *Aspergillus niger* strain GT147. The safety of Sumizyme GT was evaluated in a series of standard toxicological studies, including a 90‐day oral toxicity study in rats, an in vitro bacterial reverse mutation assay, an in vitro mammalian chromosome aberration test, and an in vivo alkaline Comet assay. Sumizyme GT was not mutagenic or genotoxic, and administration of the enzyme by gavage at doses up to 2,570 mg total organic solids (TOS)/kg body weight (bw) per day for 90 days was without any systemic toxicity. The no‐observed‐adverse‐effect level was concluded to be 2,570 mg TOS/kg bw per day, the highest dose tested. Considering that *A. niger* has an established history of safe use in the food industry and its safety in the production of food ingredients and food enzymes is well documented, the results of these studies provide further support of the safety of glutaminase from *A. niger* when used in food production.

## Graphical abstract

Glutaminase from nongenetically modified *Aspergillus niger* was evaluated in a series of toxicological tests. The results of these studies demonstrate that glutaminase is nonmutagenic and nongenotoxic, and provide further support of the safety of glutaminase from *A. niger* when used in food production.

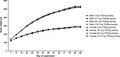

## 1 INTRODUCTION

Glutaminase (glutamine aminohydrolase; EC3.5.1.2) is an enzyme used by the food industry to produce glutamic acid‐rich food ingredients that are subsequently added to finished foods to improve the savory or “umami” flavor of foods. Traditionally, glutaminase has been used in the production of soy sauce and miso to increase the glutamate content (Harayama & Yasuhira, 1991). Glutaminase catalyzes the hydrolytic deamination of L‐glutamine to yield L‐glutamate and ammonia. The enzyme acts on the carbon–nitrogen (C‐N) bonds in linear amides and is not preferential toward C‐N bonds of peptides (Kegg, 2019). For example, glutaminase does not act upon amide groups in glutamine present in peptides, free or bound asparagine, and in certain nucleic acid bases, such as guanosine monophosphate. Glutaminase has an indirect role in imparting or enhancing the flavor profile of foods by increasing the L‐glutamate content of intermediate food ingredients. The resultant flavor profile is similar to the flavor achieved by monosodium glutamate (MSG), thereby reducing or eliminating the need for the addition of MSG to foods.

Currently, glutaminase used in food processing is typically obtained from *Bacillus amyloliquefaciens* or *Bacillus subtilis* (Amfep, 2015; Ministry of Health of the PRC, 2011; Pariza & Johnson, 2001). However, a strain of *Aspergillus niger*, a filamentous fungus, isolated from a food source has been found to have high levels of glutaminase activity, making it suitable for commercial use. *A. niger* has a well‐established history of safe use in food production, with one of the earliest reported industrial uses of *A. niger* in the production of citric acid in 1919 (Max et al., 2010). Since then, *A. niger* has been used in the production of numerous food ingredients, including chitosan and chitin‐glucan, and food enzymes, such as amylase, amyloglucosidase, asparaginase, catalase, glucose oxidase, glucanase, cellulases, lactase, lipase, inulinase, invertase, pectinase, pentosanase, phospholipase, acid proteases, and xylanases, many of which have been concluded to be generally recognized as safe by the United States Food and Drug Administration (U.S. FDA) for use in food processing (US FDA, 2011, 2012, 2018) or by other regulatory bodies (FSANZ, 2019; Health Canada, 2019).

Limited toxicological information on orally administered glutaminase was identified in the scientific literature. Ohshita et al. (2000) conducted a safety assessment of glutaminase derived from different strains of *Cryptococcus albidus*, a filamentous fungus, and reported a no‐observed‐adverse‐effect level (NOAEL) of 7.5 and 7.9 g/kg body weight (bw)/day for male and female ddY‐F mice, respectively, 8.8 and 9.9 g/kg bw per day for male and female F244/DuCrj rats, respectively, and 1.1 and 1.2 g/kg bw per day for male and female Std:Wistar rats, respectively, the highest dose tested in all cases, based on the results of 13‐week oral toxicity studies. The reported NOAEL for a 1‐year feeding study in ddY‐F mice was 13 g/kg bw per day for males and 15.5 g/kg bw per day for females, the highest dose tested. Despite the difference in fungal species, these results suggest that glutaminase derived from *A. niger* may be without toxicological effects. The objective of this study was to evaluate the safety of a glutaminase enzyme preparation derived from *A. niger*, namely Sumizyme GT, for use in food processing. Accordingly, a battery of genotoxicity and mutagenicity studies was conducted to assess the genotoxic or mutagenic potential of Sumizyme GT, including an in vitro bacterial reverse mutation assay, an in vitro mammalian chromosome aberration test, and an in vivo alkaline Comet assay. The subchronic toxicity of Sumizyme GT was evaluated in a 90‐day repeated‐dose oral toxicity study in Sprague Dawley rats. The details of these toxicological studies are outlined in detail below.

## 2 MATERIALS AND METHODS

### 2.1 Enzyme preparation

Sumizyme GT (Lot No. 150717T) was provided by Shin Nihon Chemical Co. (Anjo, Aichi, Japan) as an odorless dark brown, viscous liquid. The same manufacturing lot was used in all studies^1^. The test article used in all studies met the established product specifications based on analytical testing. The enzyme preparation contained 1,478 U/ml glutaminase activity with the amount of test article per unit of enzyme activity providing 128.5mg total organic solids (TOS) per ml. The test article was stored in airtight containers at −30 to −5°C. The test article was demonstrated analytically to be stable as validated by consistent enzyme activity and ultraviolet absorbance (280 nm) throughout the study period in the 90‐day study.

Sumizyme GT was prepared by standard culture methods with *A. niger* strain GT147. This strain of *A. niger* was selected based on its ability to produce high levels of glutaminase activity, its viability, and suitability for industrial production, including its lack of mycotoxin production. *A. niger* strain GT147 has been deposited at the Biological Resource Center, National Institute of Technology and Evaluation (Deposit No. 00326).

### 2.2 Genotoxicity studies

#### 2.2.1 Bacterial reverse mutation test (Ames test)

The bacterial reverse mutation test was conducted in accordance with Organisation for Economic Co‐operation and Development (OECD) Guideline No. 471 (OECD, 1997) using the preincubation method and a modified method (treat‐and‐wash method) that involves a washing step to remove free amino acids, such as histidine, released into the culture medium that have been associated with overall increase in bacterial growth and in additional spontaneous mutations to occur. The treat‐and‐wash method is considered to be a valid method to eliminate the potential for “false‐positive” effects due to the presence of free amino acids in the test substance (EFSA, 2014; Thompson, Morley, Kirkland, & Proudlock, 2005).

The mutagenicity of Sumizyme GT was tested in the Ames assay using *Salmonella typhimurium* strains TA100, TA1535, TA98, and TA1537 (provided by Dr. Bruce N. Ames, University of California) and *Escherichia coli* strain WP2*uvrA* (provided by the National Institute of Health Sciences, Japan). Distilled water served as the negative control. The following compounds served as the positive controls in studies conducted with the preincubation method without metabolic activation: 0.01 µg/plate 2‐(2‐furyl)‐3‐(5‐nitro‐2‐furyl)acrylamide (AF‐2) (Lot No. STQ3987, Wako Pure Chemical Industries) for TA100 and WP2*uvrA* and 0.1 µg/plate for TA98, 0.5 µg/plate sodium azide (Lot No. YSR7529, Wako Pure Chemical Industries) for TA1535, and 80 µg/plate 9‐aminoacridine hydrochloride (9‐AA) (Lot No. 09820CEV, Sigma‐Aldrich) for TA1537. In studies conducted with the preincubation method with metabolic activation, 0.5 µg/plate 2‐aminoanthracene (2‐AA) (Lot No. TLH6618, Wako Pure Chemical Industries) served as the positive control for TA98, 1.0 µg/plate for TA100, 2.0 µg/plate for TA1535 and TA1537, and 10 µg/plate for WP2*uvrA*. In the modified (treat‐and‐wash) method, AF‐2, 4‐nitroquinoline *N*‐oxide (Lot No. N4RDF, Tokyo Chemical Industry) and 9‐AA served as the positive controls without metabolic activation, while 2‐AA served as the positive control in the studies with metabolic activation. The microsomal fractions (S9) obtained from the livers of phenobarbital‐ and 5,6‐benzoflavone‐induced male Sprague Dawley rats were used for metabolic activation purposes.

Preliminary and concentration‐finding studies were conducted using the preincubation method to establish suitable concentrations and to evaluate mutagenicity prior to the initiation of the main study. A concentration‐finding study was also conducted using the treat‐and‐wash method to establish appropriate concentrations for the main study using the modified method.

In the main study using the preincubation method, Sumizyme GT concentrations were prepared using a dilution factor of 2 to obtain final test concentrations of 402, 803, 1,610, 3,210, and 6,430 µg TOS/plate. The undiluted Sumizyme GT stock solution (12,850 µg TOS/plate) was the highest concentration tested in *S. typhimurium* TA1537 in the presence of metabolic activation. *E. coli* WP2*uvrA* was tested at concentrations of 25.1, 50.2, 100, 201, 402, 803, 1,610, and 3,210 µg TOS/plate in the presence and absence of metabolic activation. All test concentrations were tested in duplicate in the modified preincubation (treat‐and‐wash) method. Sumizyme GT concentrations were tested in duplicate at 803, 1,610, 3,210, 6,430 and 12,850 µg TOS/plate in the presence and absence of metabolic activation in *S. typhimurium* TA100, TA1535, TA98 and TA1537.

Precipitation of the test article was observed macroscopically in all plates at the initiation of treatment and at the time of colony counting. Growth inhibition was examined using a stereoscopic microscope and revertant colonies counted using an automated colony analyzer (CA‐11, System Sciences). Responses were considered positive if the mean number of revertant colonies was at least 2 times greater than the negative control, a concentration‐dependent relationship was observed, or the effects were reproducible. Statistical analysis of the data was not conducted.

#### 2.2.2 In vitro mammalian chromosomal aberration test

The in vitro mammalian chromosome aberration test was conducted in accordance with OECD Test Guideline No. 473 (OECD, 2014). Cultured Chinese hamster lung (CHL) fibroblast cells (National Institute of Health Sciences, Japan) were used in either a 6‐hr short‐term assay in the presence or absence of metabolic activation or a 24‐hr continuous assay in the absence of metabolic activation. The S9 microsomal fractions were obtained as previously described for the bacterial reverse mutation test. Distilled water served as the negative control. Mitomycin C (MMC) (Lot No. 577AEE, Kyowa Hakko Kirin) served as the positive control at a concentration of 0.1 µg/ml in the short‐term assay in the absence of metabolic activation and at a concentration of 0.05 µg/ml in the continuous treatment assay. Cyclophosphamide (Lot No. 4,399, Shionogi) served as the positive control in the short‐term assay in the presence of metabolic activation at a concentration of 12.5 µg/ml.

In the short‐term assay, CHL cells were incubated with Sumizyme GT for 6 hr at final concentrations of 1,000 to 10,000 µg TOS/ml in the absence of metabolic activation and 250 to 8,000 µg TOS/ml in the presence of metabolic activation. In the continuous assay, CHL cells were incubated with Sumizyme GT for 24 hr at final concentrations of 500 to 10,000 µg TOS/ml in the absence of metabolic activation. The concentrations were chosen based on preliminary testing to determine mitotic index and cell growth inhibition (data not shown).

Microscopic examinations were carried out in the short‐term test in the absence and presence of metabolic activation and the continuous treatment test with analyzable upper‐limit Sumizyme GT concentrations of 4,000, 3,000 and 3,000 µg TOS/ml, respectively.

Precipitation of the test article and changes in the color of the culture medium were observed with the naked eye at the beginning of the study and at the end of the treatment.

The CHL cells were seeded in culture plates and incubated for 3 days, followed by treatment with the negative or positive control or Sumizyme GT. The cells were then incubated for 6 hr for the short‐term assay and 24 hr for the continuous assay. Following the incubation period, the cells in the short‐term assay were detached by an effect of the test article. The medium was removed from each plate, transferred into individual centrifuge tubes, and rinsed with Dulbecco's phosphate‐buffered saline (PBS) (Lot No. RNBF4689, Sigma‐Aldrich), and fresh medium was added to each plate. The suspension was then centrifuged (1,000 rpm, 5 min), the supernatant was removed, and the collected cells were washed with PBS. Following removal of the supernatant, fresh medium was added to each tube and the cells were resuspended and returned to the original plate. The cells were incubated for an additional 18 hr (total incubation of 24 hr after the start of exposure).

Two hours prior to preparation of the slides for examination, 0.2 µg/ml of colcemid solution (Lot No. 1,776,482; Life Technologies) was added to each plate to inhibit mitosis in the metaphase. Following incubation, the culture medium was transferred to a centrifuge tube and the cells were detached from the plate with 2 ml of 0.25% trypsin–ethylenediaminetetraacetic acid (EDTA) (Lot No. 1,798,279; Life Technologies). The cell suspension was also added to the tube. Relative cell growth rate was calculated using a portion of the cell suspension, and the remaining cell suspension was centrifuged (1,000 rpm, 5 min), and the supernatant was removed. The cells were then exposed to a hypotonic treatment for 16 min in 5 ml of a 75 mmol/L KCl solution that was prewarmed to 37°C and centrifuged, and the supernatant was removed. Next, the cells were twice fixed with an ice‐cold 3:1 mixture of methanol and acetic acid, followed by cell resuspension in the fresh fixative mixture. One chromosome slide per plate was prepared to confirm the cell density. After confirmation, each suspension was added to a slide and 3 slides per plate were prepared for chromosome analysis. The slides were stained with 1.2% Giemsa solution (Lot No. HX57878704; Merck). Slides with greater than 50% relative cell growth rates were not subjected to chromosome analysis as the slides contained the presence of many c‐mitosis figures, and showed almost no analyzable mitosis. The analyzable upper‐limit GT concentrations were therefore selected as the highest concentrations (−S9 assay: 4,000 µg TOS/ml; +S9: 3,000 µg TOS/ml; 24‐hr assay: 3,000 µg TOS/ml); in addition, three concentrations at appropriate intervals (including the highest concentration) were selected for microscopic examination.

Microscopic evaluation of the selected concentrations involved examination of 150 metaphase cells per plate or 300 metaphase cells per concentration, for the following chromosomal or chromatid‐type aberrations: chromosome and chromatid gaps, breaks, exchanges, and others. In addition, the number of polyploid cells (38 chromosomes or more) was counted by observing 300 metaphases for each concentration.

The incidence of aberrant cells was analyzed by a one‐side Fisher's exact test with a 2.5% level of significance. If significant differences were observed, a one‐sided Cochran–Armitage test with a 2.5% level of significance was used to analyze concentration dependency. Responses were considered to be positive if the incidence of cells with chromosomal aberrations was statistically significantly increased in more than one of the test concentrations compared to the negative control, a significant concentration‐dependent increase is observed, and the observed incidence is greater than the acceptable range calculated from the historical data of the negative control group. The final evaluation was made on the total incidence of aberrant cells minus the number of cells with only gaps.

#### 2.2.3 In vivo alkaline comet assay

The in vivo mammalian alkaline comet assay was conducted in accordance with OECD Test Guideline No. 489 (OECD, 2016a). Forty male Crl:CD(SD) [SPF] rats were obtained at 7 weeks of age from Charles River Laboratories Japan, Inc. Upon receipt and throughout acclimation, animals were observed for general condition and body weights were measured on the day of receipt (Day −7) and the final day of the quarantine and acclimation period (Day 1). No abnormalities in clinical signs and body weight were observed during the acclimation period (Days −7 to 1). On the initial day of dosing (Day 1), animals were randomly assigned to 1 of 6 groups (6 rats/group) based on body weights.

The doses were selected based on the results of a 2‐week repeated‐dose oral toxicity study of Sumizyme GT in rats, in which no toxicity was observed at the highest tested dose of 2,570 mg TOS/kg. The low‐ and mid‐dose formulations were prepared by serially diluting the Sumizyme GT stock solution (128.5 mg TOS/ml) with distilled water to obtain concentrations of 32.1 and 64.3 mg TOS/ml, equivalent to doses of 643 and 1,285 mg TOS/kg. The undiluted stock solution (128.5 mg TOS/ml or 2,570 mg TOS/kg) served as the highest tested dose. Distilled water served as the negative control. The test article and vehicle control were administered at a dosing volume of 20 ml/kg bw per day *via* oral gavage using a plastic syringe and Teflon gastric tube. Ethyl methanesulfonate (Lot No. BCBN1209V, Sigma‐Aldrich) served as the positive control at a dose of 200 mg/kg. The positive control was administered by gavage using a plastic syringe and Teflon gastric tube at a dosing volume of 10 ml/kg. The test article and negative and positive controls were administered once daily for 2 consecutive days at 21‐hr intervals.

All animals were observed for clinical signs at 0.5, 21 (prior to second administration), 21.5, and 24 hr (prior to necropsy) after the first administration. Body weight was measured prior to necropsy. The animals were euthanized by inhalation of CO_2_ 3 hr after the second administration of either test article, negative control, or positive control. The stomach (glandular stomach) and duodenum were resected and rinsed with homogenizing buffer prepared from Hanks’ balance salt solution (Lot No. 1,843,038; Life Technologies) containing 8.93 g/L EDTA·2Na (Lot No. KP001, Dojindo Laboratories) and 10 vol% dimethyl sulfoxide (Lot No. DSG2253, Wako Pure Chemical Industries), pH 7.5, and macroscopically observed for abnormalities. A portion of the glandular stomach and duodenum (approximately 1 cm from the pylorus) were collected for potential histopathological examination, and the remaining tissues were used for the comet assay.

For the comet assay, the glandular stomach was dissected and incubated with cooled homogenizing buffer for 15 to 30 min. The surface epithelium was gently scraped with a blade and discarded; the epithelial cells were released following scraping with homogenizing buffer. The duodenum was dissected and rinsed with homogenizing buffer. An adequate volume of homogenizing buffer was added to each of the tissue samples, and the samples were homogenized using a Dounce tissue grinder. The cells were then centrifuged (800 rpm, 5 min), the supernatant was removed, and the cells were resuspended in the remaining supernatant. Each suspension (10 µl) was put into a microbe tube. Slides were prepared in triplicates (2 slides for evaluation, 1 for spare) on a superfrosted glass slide precoated with 1.0% agarose gel. Ninety microliters of 0.5% low‐melting agarose gel (Lot No. 0,000,587,240; Lonza Rockland) was added to the microbe tubes containing cells and mixed. Following mixing, 90 µl of the cell‐agarose mixture was placed on the precoated slide and covered with a noncoated superfrosted glass slide. After the agarose solidified and the covered glass slide was removed, the slides were placed in lysing solution (pH 10) and left overnight under refrigerated and light‐protected conditions. The slides were then rinsed with electrophoresis buffer and placed in a submarine‐type electrophoresis chamber (BE‐540, BIOCRAFT), and chilled electrophoresis buffer was gently added to the chamber until the slides were completely immersed and the slides were left for 20 min (unwinding). Electrophoresis was conducted at a constant voltage of 25 V (0.7 V/cm; initial current: 300 mA) for 20 min. Throughout the unwinding and electrophoresis steps, the electrophoresis chamber was cooled on ice in order to keep the electrophoresis buffer at a low temperature (at the start of unwinding: 0.1 to 0.6°C, at the start of electrophoresis: 1.2 to 2.4°C, at the end of electrophoresis: 3.5 to 5.1°C). Following electrophoresis, neutralization of the alkali in the gels was carried out by immersion of the slides into a neutralizing solution (pH 7.5) for 10 min. The neutralized slides were dehydrated for 10 min in ethanol (≥99.5%), and no damage was observed; thus, the spare slides were discarded without electrophoresis.

Prior to microscopic examination, all slides were coded at random and treated with 50 µl of SYBR^®^ Gold nucleic acid gel stain (Lot No. 1,832,446; Life Technologies) diluted 5,000‐fold with Tris‐EDTA buffer (pH 8.0; Lot No. 02234G; Nippon Gene). The images of DNA migration of cells were examined with a fluorescence microscope (objective: ×20) with IB excitation. The images were then imported to a computer through a CCD camera that is attached to the microscope. A Comet assay analyzer (Comet Assay IV system, Perceptive Instruments) was used for the analysis.

One‐hundred fifty cells [75 cells per slide; that is, 750 cells per group (5 animals)] were analyzed using a fluorescence microscope (×200), and the number of hedgehogs was counted. Histopathological examination of the stomach or duodenum was not conducted as no clear DNA damage was observed in the comet assay. The percentage of DNA in the tail relative to the total (% tail DNA:Tail % intensity) served as the indicator for DNA damage. Each slide was analyzed to determine the media % tail DNA (slide value), and the mean of the slide values for each animal (animal value) was calculated. The % tail DNA (animal value) was logarithmically transformed prior to statistical analysis. A two‐sided Dunnett's multiple comparison test was used to analyze the % tail DNA (mean of animal values) between the negative control and each test article‐treated group at a 5% level of significance. If a significant difference was observed, a two‐sided linear trend test was conducted to analyze dose dependence at a 5% level of significance. A one‐sided Aspin–Welch's *t* test was used to analyze the % tail DNA (mean of animal values) for the comparison between the negative and positive control groups at a 2.5% level of significance. The test result was considered to be positive, if at least 1 test article‐treated group demonstrates a statistically significant increase in % tail DNA compared to the control, a significant dose dependence is observed in the % tail DNA, and the % tail DNA (mean of animal values) of the test article‐treated groups exceeds the historical data of the negative control group.

A second comet assay was conducted to confirm the results of Sumizyme GT in the duodenum of rats at doses of 1,260, 1,800, and 2,570 mg TOS/kg. The dose formulations were prepared as previously described. A second group with heat‐inactivated Sumizyme GT was also tested at the highest dose. Following euthanization of the animals, the duodenum was resected as it is immediately exposed to the test article following oral administration and the test article is considered to be present at a relatively high concentration in this organ. The duodenum was dissected, rinsed, and prepared for microscopic examination as previously described. Statistical analysis was conducted using a two‐sided Dunnett's multiple comparison test. As no significant difference was observed, dose dependence (trend) was not analyzed. Following the analysis of the % tail DNA (mean of animal values) using the one‐sided Aspin–Welch's *t* test, a two‐sided *F* test was used to analyze the % tail DNA (mean of animal values) for homogeneity of variance of the negative control and inactivated Sumizyme GT groups and between the activated and inactivated Sumizyme GT high‐dose groups at a level of significance of 5%. Since homogeneity of variance was determined to not be significant by the *F* test, a one‐sided Student's *t* test was used to compare the same groups at a level of significance of 2.5%.

### 2.3 90‐day repeated‐dose toxicity study

The 90‐day repeated‐dose oral toxicity study was conducted in accordance with OECD Test Guideline No. 408 (OECD, 1998b).

#### 2.3.1 Preparation of dosing formulations

The low‐dose and mid‐dose formulations were prepared by fourfold serial dilution of the thawed Sumizyme GT stock solution (128.5 mg TOS/ml) with distilled water to obtain concentrations of 8.0 and 32.1 mg TOS/ml, equivalent to doses of 161 and 643 mg TOS/kg bw per day, respectively. The undiluted stock solution (128.5 mg TOS/ml) served as the high‐dose formulation, equivalent to 2,570 mg TOS/kg bw per day. The stability of the dose formulations was validated by measurements of enzyme activity, and the results demonstrated the test doses were stable after storage under refrigeration temperatures for 7 days followed by 24‐hr storage at room temperature at concentrations up to 128.5 mg TOS/ml.

#### 2.3.2 Animals and treatment

Forty‐five male and 45 female 4‐week‐old Crl:CD(SD) [SPF] rats were obtained from Charles River Laboratories Japan, Inc. Upon receipt, animals were observed daily for general condition throughout the 7‐day acclimation period and body weight was measured on the day of receipt (Day −7) and the final day of the quarantine and acclimation period (Day 1). No abnormalities in clinical signs and body weight were observed from Days −7 to 1. Before dosing (Days −2 and −1), the animals underwent ophthalmological examination. Unignorable congenital defects were observed in the eyes of 1 male and 2 females; these rats were excluded from the study prior to group assignment (surplus animals). On the first day of dosing (Day 1), animals were randomly assigned to groups (10/sex/group) based on body weights using a computer system package for safety studies (LATOX‐F/V5, FFC). The body weight of the animals used in the study ranged from 129 to 155 g for males and 111 to 135 g for females.

The doses of Sumizyme GT were selected based on the results of a 14‐day dose range‐finding study in rats in which no treatment‐related toxicological effects on general condition, body weight change, food consumption, hematological parameters, and necropsy findings were reported at doses up to 2,570 mg TOS/kg bw per day (data not shown). As a result, the high‐dose group was selected as the maximum dose and 161 and 643 mg TOS/kg bw per day were selected for the low‐ and mid‐dose levels, respectively, in the 90‐day study. The control animals received distilled water (vehicle solvent) at an equivalent dosing volume to the treatment group. The test article and the vehicle control were administered at a dosing volume of 20 ml/kg bw per day *via* gavage using a plastic syringe and Teflon gastric tube. The dosing solutions were prepared once or more within 7 days at concentrations of 8.0, 32.1, and 128.5mg TOS/ml for the low‐dose (161 mg TOS/kg bw per day), mid‐dose (643 mg TOS/kg bw per day), and high‐dose (2,570 mg TOS/kg bw per day) groups, respectively.

#### 2.3.3 Clinical observations, body weights, food consumption, and ophthalmology

The general condition of the animals was observed and recorded twice per day (before and after dosing) and once before necropsy (Day 91). Tests for sensorimotor function, grip strength, and locomotor activity were conducted in all animals near the end of the dosing period. Detailed observations were conducted prior to dosing and once a week thereafter and included home cage observation, responses on removal from cage, and behavior in open field. Following the other functional observation battery (FOB) tests, animals were immediately tested for individual locomotor activities using a cage‐stationary‐type activity‐measurement system (LOCOMO LS‐7, Melquest) every minute for 1 hr. Body weight of the animals was measured every 7 days from the initiation of the study (Day 1) until Day 85, just prior to necropsy (Day 90), and on the day of necropsy (Day 91). Body weight gain was calculated from Day 1 to 90. The food amounts were weighed on the days of body weight measurement, and the remaining food was measured on the subsequent body weight measurement day. Mean daily food consumption (g/day) was calculated for individual animals based on the difference of amounts of the food. Ophthalmological examinations were carried out in all the animals during the quarantine and acclimation period (Days −2 and −1) and the final week of the dosing period (Day 86). Examinations included observations for appearance and light reflex and the anterior part of the eyeballs, optic media, and fundus oculi following pupil dilation (Lot No. M514151; Mydrin P®, Santen Pharmaceutical).

#### 2.3.4 Clinical pathology and urinalysis

Hematological and blood chemical examinations were conducted in all surviving animals on the day of the scheduled necropsy (Day 91). Prior to necropsy, the animals were fasted overnight, and blood samples were collected the following day from the abdominal aorta under isoflurane anesthesia. Hematological examination was carried out using blood and plasma samples. Blood samples were collected in tubes containing an anticoagulant (EDTA dipotassium salt) and were analyzed with a Hematology System (ADVIA120; Bayer). The following parameters were analyzed: hematocrit, hemoglobin, red blood cell count, mean corpuscular volume, mean corpuscular hemoglobin, mean corpuscular hemoglobin concentration, reticulocyte ratio, reticulocyte count, platelet count, white blood cell count, differential leukocyte ratios, leukocyte count (neutrophil, lymphocyte, monocyte, eosinophil, basophil),and large unstained cell count.

Plasma samples were collected in tubes containing an anticoagulant (3.2% sodium citrate solution) and subjected to centrifugation at 1,700×*g* for 13 min at room temperature. The plasma samples were analyzed with a coagulation analyzer (STA Compact, Roche), and the following hematological parameters were evaluated: prothrombin time and activated partial thromboplastin time.

Serum samples were obtained from blood samples and collected into tubes containing a Gel and Clot activator (Venoject II, Terumo) for blood chemistry examination. The samples were subjected to centrifugation at 1,700×*g* for 7 min at room temperature and analyzed using an automatic analyzer (Hitachi 7,170, Hitachi). The following parameters were evaluated: total protein, glucose, triglyceride, total cholesterol, blood urea nitrogen, creatinine, total bilirubin, aspartate aminotransferase, alanine aminotransferase, γ‐glutamyl transpeptidase, calcium, and inorganic phosphorus. The samples were also analyzed using an electrolyte analyzer (EA06R, A&T) to examine concentrations of sodium, potassium, and chloride. An electrophoresis analyzer (Epalyzer 2 plus, Helena Laboratories) was used to analyze the following additional blood chemistry parameters: albumin ratio, α_1_‐globulin ratio, α_2_‐globulin ratio, β‐globulin ratio, γ‐globulin ratio, albumin/globulin ratio, albumin concentration, α_1_‐globulin concentration, α_2_‐globulin concentration, β‐globulin concentration, and γ‐globulin concentration.

Urine samples were collected within 3 hr after urination (fresh urine) and 24 hr after urination (pooled urine) for urinalysis. The animals remained fed and were supplied with water during urine sample collections. Fresh urine was analyzed using Ames test strips (N‐Multistix, SG‐L, Siemens Healthcare Diagnostics) and an automatic strip reader (CLINITEK Advantus, Siemens Healthcare Diagnostics) for the following parameters: pH, occult blood, ketone bodies, glucose, protein, bilirubin, and urobilinogen. Following confirmation of volume and color of urine, the 24‐hr urine samples were centrifuged at 400×*g* for 5 min. The supernatant was analyzed for electrolyte concentrations (sodium, potassium, and chloride) using an electrolyte analyzer (EA06R) and osmotic pressure using an osmotic pressure analyzer (AUTO&STAT ™ OM‐6030, Arkray Factory). Urinary volume was also used to calculate the total excretion value of each electrolyte. Urinary sediments were stained by the Sternheimer method and examined microscopically.

#### 2.3.5 Pathology

Pathological examinations consisted of organ weight measurements, macroscopic examination (necropsy), and histopathological examination. The following organs of all animals were weighed: brain, heart, liver, kidneys, spleen, testes, adrenal glands, ovaries, thymus, uterus, and epididymides. The organ‐to‐body weight ratio (relative organ weight; based on body weight measured on the day of necropsy) and absolute organ weight (absolute organ weight/final body weight × 100) were calculated. The organs and tissues preserved for histopathological examination from all animals in all groups are consistent with those specified in the OECD guidelines. The testes were prefixed in formalin–acetic acid solution, and the eyes (including optic nerve and Harderian glands) were prefixed in Davidson's solution, followed by fixation in 10% neutral buffered formalin solution. The lungs (left and right) were fixed by dropping infusion of fixative. All other organs and tissues were fixed in an adequate volume of 10% neutral buffered formalin solution. All the fixed organs and tissues were embedded in paraffin, sectioned, and stained with hematoxylin and eosin prior to histopathological examination. Microscopic examination included specimens from the control and high‐dose group only as no treatment‐related findings were observed at the high‐dose level. Types and severity of all histopathological findings were recorded.

#### 2.3.6 Statistical analysis

Analysis of the body weight, body weight gain, food consumption, metric FOB data (grip strength and locomotor activity), hematology, blood chemistry, urinalysis (volume, osmotic pressure, electrolytes), and absolute and relative organ weights of the animals were analyzed by the Bartlett's test for equality of variance. Data determined to be homogenous based on Bartlett's test were analyzed using Dunnett's multiple comparison test to evaluate the statistically significant differences between the control group and test substance‐treated group. Data determined to be heterogenous based on Bartlett's test were analyzed using Steel's test to evaluate the statistical significance between the control group and test substance group. The significance of Bartlett's test was analyzed at the 5% level of significance, and the other 2 tests were analyzed at the 5% and 1% level of significance using a two‐sided analysis.

## 3 RESULTS

### 3.1 Genotoxicity studies

#### 3.1.1 Bacterial reverse mutation test (Ames test)

The number of revertant colonies increased 2 times or more relative to the negative controls in strains TA100, TA1535, TA98, and TA1537 in the presence and absence of metabolic activation in the preliminary study and in the absence of metabolic activation in the concentration‐finding study using the preincubation method (Table 1). The number of revertant colonies also increased 2 times or more in the concentration‐finding study in strains TA100, TA1535, and TA98 in the presence of metabolic activation relative to the negative controls. Accelerated growth in background bacteria with a cloudy medium was observed at 1,290 and 12,850 µg TOS/plate and 1,430, 4,289 and 12,850 µg TOS/plate in the preliminary and concentration‐finding studies, respectively, in all strains and in both assays. Bacterial growth inhibition was also observed at 1,290 and 1,430 µg TOS/plate in the preliminary and concentration‐finding studies, respectively, in strain WP2*uvrA* and in both assays.

**Table 1:** Results of concentration‐finding bacterial reverse mutation test of GT using preincubation method

Concentration (µg TOS/plate)	Revertant colonies per plate (mean ± *SD*)									
	*Salmonella typhimurium*								*Escherichia coli*	
	TA100		TA1535		TA98		TA1537		WP2*uvrA*	
	−S9	+S9	−S9	+S9	−S9	+S9	−S9	+S9	−S9	+S9
Negative control (distilled water)^a^	117 ± 7	120 ± 5	13 ± 1	13 ± 1	24 ± 6	26 ± 2	10 ± 3	14 ± 1	29 ± 3	21 ± 4
1.96	–	–	–	–	–	–	–	–	24 ± 3	20 ± 1
5.88	–	–	–	–	–	–	–	–	22 ± 3	22 ± 2
17.6	–	–	–	–	–	–	–	–	21 ± 5	23 ± 4
52.9	110 ± 8	125 ± 10	8 ± 2	11 ± 3	26 ± 4	30 ± 3	11 ± 3	19 ± 3	23 ± 3	22 ± 2
159	124 ± 8	121 ± 9	11 ± 2	11 ± 2	24 ± 3	34 ± 3	12 ± 3	19 ± 5	27 ± 2	26 ± 2
476	130 ± 4	141 ± 6	14 ± 2	10 ± 2	28 ± 3	33 ± 3	10 ± 2	13 ± 2	25 ± 3	23 ± 1
1,430	143 ± 9	140 ± 15	15 ± 3	10 ± 1	32 ± 6	46 ± 6^d^	11 ± 2^d^	23 ± 2^d^	24 ± 2^e^	21 ± 3^d^, ^e^
4,280	182 ± 4^d^	215 ± 10^d^	22 ± 2^d^	24 ± 3^d^	41 ± 5^d^	66 ± 12^d^	9 ± 3 d	20 ± 3^d^	34 ± 2^d^, ^e^	24 ± 4^d^, ^e^
12,850	291 ± 24^d^	296 ± 37^d^	33 ± 6^d^	35 ± 3^d^	77 ± 3^d^	95 ± 21^d^	22 ± 3^d^	21 ± 4^d^	–	–
Positive control^b^, ^c^	637 ± 32	785 ± 25	552 ± 27	323 ± 13	704 ± 31	357 ± 14	200 ± 5	186 ± 16	105 ± 11	716 ± 31

Abbreviations: −S9, in the absence of S9; +S9, in the presence of S9; 2‐AA, 2‐aminoanthracene; 9‐AA, 9‐aminoacridine hydrochloride; AF‐2, 2‐(2‐furyl)‐3‐(5‐nitro‐2‐furyl) acrylamide; GT, glutaminase from nongenetically modified *A. niger* strain GT147; NaN_3,_ sodium azide; *SD*, standard deviation; TOS, total organic solids.

a 100 µl/plate.

b Positive control −S9: TA100 and WP2*uvrA* = 0.01 µg/plate AF‐2; TA1535 = 0.5 µg/plate NaN_3_; TA98 = 0.1 µg/plate AF‐2; TA1537 = 80 µg/plate 9‐AA.

c Positive control + S9: TA100 = 1.0 µg/plate 2‐AA; TA1535 and TA1537 = 2.0 µg/plate 2‐AA; TA98 = 0.5 µg/plate 2‐AA; WP2*uvrA* = 10 µg/plate 2‐AA.

d The growth of background lawn of bacteria was accelerated, and the plates looked turbid.

e Growth inhibition was observed.

In the main study conducted using the preincubation method, the number of revertant colonies increased 2 times or more in strain TA1537 at the highest concentration tested (12,850 µg TOS/plate) in the presence of metabolic activation relative to the negative control (Table 2). The number of revertant colonies was comparable to that of the negative control in *E. coli* strain WP2*uvrA* in the presence and absence of metabolic activation. Accelerated growth in background bacteria with a cloudy medium and bacterial growth inhibition was observed in WP2*uvrA* at the 2 highest concentrations (1,610 and 3,210 µg TOS/plate) in the presence and absence of metabolic activation and in strain TA1537 at concentrations of 803, 1,610, 3,210, 6,430 and 12,850 µg TOS/plate in the presence of metabolic activation (Table 2).

**Table 2:** Results of the main bacterial reverse mutation test of GT using the preincubation method

Concentration (µg TOS/plate)	Revertant colonies per plate (mean ± *SD*)		
	*Escherichia coli* WP2*uvrA*		*Salmonella typhimurium* TA1537
	−S9	+S9	+S9
Negative control (distilled water)^a^	28 ± 4	25 ± 3	14 ± 1
25.1	24 ± 3	28 ± 4	–
50.2	29 ± 5	26 ± 2	–
100	24 ± 3	26 ± 6	–
201	30 ± 2	31 ± 5	–
402	26 ± 5	31 ± 5	14 ± 3
803	26 ± 4	24 ± 3^d^	15 ± 5^e^
1,610	28 ± 4^d^, ^e^	30 ± 3^d^, ^e^	18 ± 0^e^
3,210	29 ± 4^d^, ^e^	28 ± 2^d^, ^e^	16 ± 3^e^
6,430	–	–	18 ± 4^e^
12,850	–	–	35 ± 5^e^
Positive control^b^, ^c^	125 ± 6	994 ± 142	179 ± 34

Abbreviations: –, not applicable; −S9, in the absence of S9; +S9, in the presence of S9; 2‐AA, 2‐aminoanthracene; AF‐2, 2‐(2‐furyl)‐3‐(5‐nitro‐2‐furyl) acrylamide; GT, glutaminase from nongenetically modified *A. niger* strain GT147; *SD*, standard deviation; TOS, total organic solids.

a 100 µl/plate.

b Positive control −S9: WP2*uvrA* = 0.01 µg/plate AF‐2.

c Positive control + S9: TA1537 = 2.0 µg/plate 2‐AA; WP2*uvrA* = 10 µg/plate 2‐AA.

d Growth inhibition was observed.

e The growth of background lawn of bacteria was accelerated, and the plates looked turbid.

In the main and concentration‐finding studies using the modified (treat‐and‐wash) method, the number of revertant colonies was comparable to that of the negative control in strains TA100, TA1535, TA98, and TA1537 in the presence and absence of metabolic activation (Table 3). No accelerated growth in background bacteria and bacterial growth inhibition was observed at any concentration in the assays (main and concentration‐finding). As expected, the number of revertant colonies in the positive control increased 2 times or more relative to the negative control. No precipitation of the test article was observed at the start of exposure and at the time of colony counting. The mean numbers of revertant colonies in the negative and positive control groups were all within the acceptable range from historical data, confirming the validity of the study (Table 3).

**Table 3:** Results of main bacterial reverse mutation test of GT using the treat‐and‐wash method

Concentration (µg TOS/plate)	Revertant colonies per plate (mean ± *SD*)							
	TA100		TA1535		TA98		TA1537	
	−S9	+S9	−S9	+S9	−S9	+S9	−S9	+S9
Negative control (distilled water)^a^	136 ± 9	150 ± 16	13 ± 3	22 ± 2	40 ± 3	57 ± 4	5 ± 1	17 ± 3
803	140 ± 6	153 ± 9	11 ± 0	13 ± 3	40 ± 3	46 ± 4	6 ± 2	21 ± 3
1,610	145 ± 7	152 ± 4	8 ± 3	13 ± 3	37 ± 0	51 ± 2	6 ± 2	14 ± 2
3,210	131 ± 8	131 ± 7	11 ± 2	17 ± 2	34 ± 2	43 ± 2	4 ± 1	13 ± 2
6,430	124 ± 5	145 ± 8	13 ± 1	13 ± 1	43 ± 5	45 ± 3	7 ± 2	15 ± 2
12,850	118 ± 9	135 ± 6	14 ± 4	17 ± 3	48 ± 5	42 ± 4	7 ± 1	13 ± 2
Positive control^b^, ^c^	700 ± 59	412 ± 30	62 ± 4	132 ± 11	302 ± 11	239 ± 27	554 ± 19	91 ± 4

Abbreviations: −S9, in the absence of S9; +S9, in the presence of S9; 2‐AA, 2‐aminoanthracene; 4‐NQO, 4‐nitroquinoline 1‐oxide; 9‐AA, 9‐aminoacridine hydrochloride; AF‐2, 2‐(2‐furyl)‐3‐(5‐nitro‐2‐furyl) acrylamide; GT, glutaminase from nongenetically modified *A. niger* strain GT147; *SD*, standard deviation; TOS, total organic solids.

a 100 µl/plate.

b Positive control −S9: TA100 = 0.02 µg/plate AF‐2; TA1535 = 0.5 µg/plate 4‐NQO; TA98 = 0.05 µg/plate AF‐2; TA1537 = 5.0 µg/plate 9‐AA.

c Positive control + S9: TA100 = 1.0 µg/plate 2‐AA; TA1535 and TA1537 = 2.0 µg/plate 2‐AA; TA98 = 0.5 µg/plate 2‐AA.

#### 3.1.2 In vitro mammalian chromosomal aberration test

In the short‐term assay in the absence of metabolic activation, the incidence of cells with structural chromosomal aberrations was 2.7%, 5.0%, and 4.7% at Sumizyme GT concentrations of 1,000, 3,000, and 4,000 µg TOS/ml, respectively, and a significant (*p* ≤ .025) concentration‐dependent increase was observed (Table 4). The incidences at 3,000 and 4,000 µg TOS/ml were significantly higher compared to the negative control (0.7%). The incidence of polyploid cells treated with the test article was 0.3%, 0.7%, and 0.3% at Sumizyme GT concentrations of 1,000, 3,000 and 4,000 µg TOS/ml, respectively; however, statistical significance was not observed compared to the negative control group (0.3%). In the presence of metabolic activation, the incidence of cells with structural chromosome aberrations were 2.7%, 7.7%, and 11.7% at Sumizyme GT concentrations of 500, 1,000, and 3,000 µg TOS/ml, respectively, and a significant (*p* ≤ .025) concentration‐dependent increase was observed (Table 4). The incidence even at 500 µg TOS/ml was significantly higher compared to the negative control (0.3%). The incidence of polyploid cells treated with the test substance was comparable to the negative control group (0.0%). The relative cell growth rates at the highest concentration in the presence (3,000 µg TOS/ml) and absence (4,000 µg TOS/ml) of metabolic activation were 71.5% and 72.8%, respectively. Microscopic examination was impossible at concentrations of 4,000 µg TOS/ml or more and 5,000 µg TOS/ml or more in the presence and absence of metabolic activation, respectively, due to an abundance of c‐mitosis figures. The positive control group (MMC) showed a high incidence of cells with structural chromosomal aberrations in the absence and presence of metabolic activation (57.3% and 71.3%, respectively) and was significantly higher compared to the negative control group.

**Table 4:** In vitro mammalian chromosome aberration test conducted with GT

Concentration (µg TOS/ml)	Relative cell growth (%)	Number of cells with structural aberrations						Number of cells with aberrations—gap (%)	Number of polyploid cells (%)
		Gap	ctb	cte	csb	cse	Others		
6‐hr short‐term treatment: −S9									
Negative control (100 µl/ml distilled water)	100.0	0	2	0	0	0	0	2 (0.7)*	1 (0.3)
1,000	92.4	1	7	1	0	0	0	8 (2.7)	1 (0.3)
3,000	79.8	4	8	7	0	0	0	15 (5.0)**	2 (0.7)
4,000	72.8	1	11	3	0	0	0	14 (4.7)**	1 (0.3)
5,000	60.3^a^	–	–	–	–	–	–	–	–
Positive control (0.1 µg/ml MMC)	79.0	10	87	130	0	0	0	172 (57.3)**	1 (0.3)
6‐hr short‐term treatment: +S9									
Negative control (100 µl/ml distilled water)	100.0	1	1	0	0	0	0	1 (0.3)*	0 (0.0)
500	97.4	0	2	6	0	0	0	8 (2.7)**	0 (0.0)
1,000	76.1	1	13	10	1	0	0	23 (7.7)**	0 (0.0)
2,000	63.3	–	–	–	–	–	–	–	–
3,000	71.5	1	26	11	0	0	0	35 (11.7)**	0 (0.0)
4,000	76.6^a^	–	–	–	–	–	–	–	–
Positive control (12.5 µg/ml CP)	45.3	3	76	196	0	0	0	214 (71.3)**	2 (0.7)
24‐hr continuous treatment									
Negative control (100 µl/ml distilled water)	100.0	1	1	1	0	0	0	2 (0.7)*	1 (0.3)
500	86.6	2	19	4	0	0	0	22 (7.3)**	2 (0.7)
1,000	96.9	7	32	7	0	0	0	39 (13.0)**	1 (0.3)
3,000	78.0	9	44	16	0	0	0	59 (19.7)**	3 (1.0)
4,000	55.8	–	–	–	–	–	–	–	–
Positive control (0.05 µg/ml MMC)	64.7	6	66	80	0	0	0	129 (43.0)**	0 (0.0)

Abbreviations: ‐gap, total number of cells with aberrations except gap; –, not applicable; −S9, in the absence of S9; +S9, in the presence of S9; CP, cyclophosphamide; csb, chromosome break; cse, chromosome exchange; ctb, chromatid break; cte, chromatid exchange; GT, glutaminase from nongenetically modified *A. niger* strain GT147; MMC, mitomycin C; TOS, total organic solids.

a There were a lot of c‐mitosis figures, and almost none of the analyzable mitosis were observed.

* Significant correlation with dosage levels (Cochran‐Armitage trend test): *p* ≤ .025.

** Significant difference from control (Fisher's exact test): *p* ≤ .025.

In the continuous assay, the incidence of cells with structural chromosomal aberrations were 7.3%, 13.0%, and 19.7% at Sumizyme GT concentrations of 500, 1,000, and 3,000 µg TOS/ml, respectively, and a significant (*p* ≤ .025)concentration‐dependent increase was observed (Table 4). At Sumizyme GT concentrations of 500 µg TOS/ml or more, the incidence of aberrations was significantly higher than that of the negative control group (0.7%). The incidence of polyploid cells treated with the test substance was 0.7%, 0.3%, and 1.0% at Sumizyme GT concentrations of 500, 1,000, and 3,000 µg TOS/ml, respectively; however, they were not statistically significantly different compared to the negative control group. The relative cell growth rate at the highest concentration (3,000 µg TOS/ml) was 78.0%. Microscopic examination was impossible at concentrations of 4,000 µg TOS/ml or more as a result of substantial c‐mitosis figures. The positive control group (MMC) showed a high incidence of cells with structural chromosomal aberrations (43.0%) and was significantly higher compared to the negative control group.

#### 3.1.3 In vivo alkaline comet assay

The in vivo alkaline comet assay was conducted to provide data to assist in the interpretation of the in vitro chromosome aberration assay and to assess potential effects due to enzyme activity (i.e., inactivated vs. active enzyme). In both tests, no macroscopic treatment‐related findings were observed in the stomach or duodenum (data not shown). No clinical signs of toxicity were observed in the treated animals, and no apparent suppression in body weight gain was observed. No apparent increases were observed in the frequencies of hedgehogs in all Sumizyme GT‐treated groups in both the stomach and duodenum cells compared with the negative control group (Tables 5 and 6). In the first comet assay, no significant increases in the mean % tail DNA in the stomach of all Sumizyme GT‐treated groups (3.55, 4.14, and 4.87) and in the duodenum of the low‐dose (643 mg TOS/kg) and mid‐dose (1,285 mg TOS/kg) Sumizyme GT‐treated groups (1.49 and 1.89) compared to the negative control group were observed (Table 5). A significant increase (2.69) was observed in the duodenum of high‐dose (2,570 mg TOS/kg) animals; this finding was significantly dose‐dependent. In the confirmatory comet assay, no significant increases were observed in the mean % tail DNA in the duodenum of all Sumizyme GT‐treated groups relative to the negative control group, including the inactivated Sumizyme GT group (Table 6). In both tests, a significant increase in the mean % tail DNA in the positive control group was observed in the stomach and duodenum cells, while the mean % tail DNA in the negative control group was within the acceptable ranges calculated from the test facility's historical data.

**Table 5:** Results of the in vivo mammalian alkaline comet assay conducted in male rats orally administered GT once daily for 2 days

Substance Dose (mg TOS/kg)	Number of animals	Stomach		Duodenum	
		% tail DNA^a^ (mean ± *SD*)	Frequency of hedgehogs (%) (mean ± *SD*)	% tail DNA^a^ (mean ± *SD*)	Frequency of hedgehogs (%) (mean ± *SD*)
Negative control (distilled water; 20 ml/kg)	5	4.10 ± 1.41	0.3 ± 0.6	1.72 ± 0.39*	0.4 ± 0.4
643	5	3.55 ± 1.07	0.8 ± 0.7	1.49 ± 0.11	0.7 ± 0.8
1,285	5	4.14 ± 2.04	0.7 ± 0.8	1.89 ± 0.17	0.9 ± 1.0
2,570	5	4.87 ± 2.32	0.5 ± 0.6	2.69 ± 0.60**	0.5 ± 0.9
Positive control (EMS; 200 mg/kg)	5	27.71 ± 2.08	1.2 ± 0.3	26.98 ± 2.30***	1.9 ± 0.6

Abbreviations: EMS, ethyl methanesulfonate; GT, glutaminase from nongenetically modified *A. niger* strain GT147; *SD*, standard deviation; TOS, total organic solids.

a The mean % tail DNA in each group was calculated from that in each animal based on the median % tail DNA in each slide.

* Significant correlation with dosage levels (linear trend test, *p* ≤ .05).

** Significantly different from negative control (Dunnett's test, *p* ≤ .05).

*** Significantly different from negative control (Aspin–Welch's *t* test, *p* ≤ .025).

**Table 6:** Results of the confirmative in vivo mammalian alkaline comet assay conducted in male rats orally administered GT once daily for 2 days

Substance Dose (mg TOS/kg)	Number of animals	Duodenum	
		% tail DNA^a^ (mean ± *SD*)	Frequency of hedgehogs (%) (mean ± *SD*)
Negative control (distilled water; 20 ml/kg)	5	1.70 ± 0.24	0.5 ± 1.2
1,260	5	1.96 ± 0.40	0.4 ± 0.4
1,800	5	1.94 ± 0.55	0.8 ± 1.1
2,570	5	1.76 ± 0.21	0.4 ± 0.6
2,570^b^		1.73 ± 0.16	0.7 ± 0.7
Positive control (EMS; 200 mg/kg)	5	26.34 ± 1.56*	2.1 ± 1.7

Abbreviations: EMS, ethyl methanesulfonate; GT, glutaminase from nongenetically modified *A. niger* strain GT147; *SD*, standard deviation; TOS, total organic solids.

a The mean % tail DNA in each group was calculated from that in each animal based on the median % tail DNA in each slide.

b Inactivated enzyme.

* Significantly different from negative control (Aspin–Welch's *t* test, *p* ≤ .025).

### 3.2 90‐day repeated‐dose toxicity study

#### 3.2.1 Clinical observations, body weights, food consumption, and ophthalmology

Throughout the administration period, no deaths or toxicological effects related to the test article treatment were observed in any of the groups. In the mid‐dose group (643 mg TOS/kg bw per day), 1 male rat experienced trauma of the neck from Days 13 to 27. No treatment‐related findings were observed in the detailed observations, frequencies of defecation and urination, and grip strength in any of the dose groups and in either sex throughout the administration period. Significant decreases were observed in the numbers of defecation in high‐dose males on Day 20 compared to the control group.

Significantly lower grip strength of hind limbs in the low‐dose (161 mg TOS/kg bw per day) and mid‐dose females was observed compared to the control group. Total amount of movement in high‐dose males was significantly lower within 0 to 10 min after the start of measurement of locomotor activity compared to the control group. No significant differences were observed in other measurement time intervals and reactivity tests in the control and test groups.

**Figure 1:**
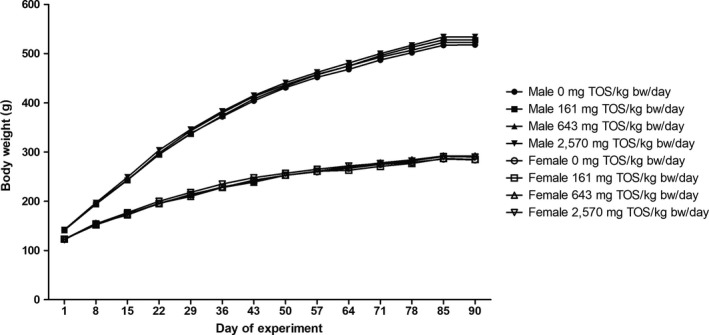
Body weight of male and female rats administered GT by gavage for 13 weeks

**Figure 2:**
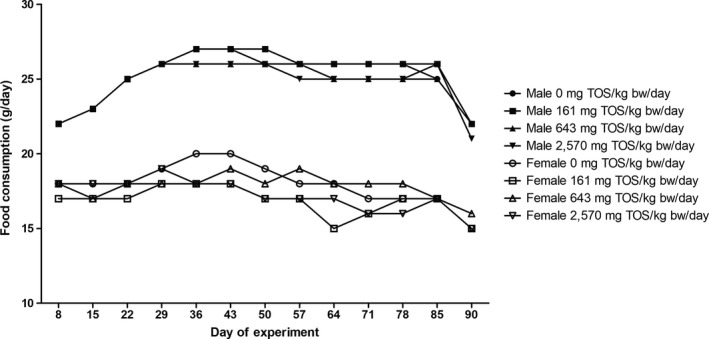
Food consumption of male and female rats administered GT by gavage for 13 weeks

No significant differences in body weights and body weight gains (Figure 1) or food consumption (Figure 2) were observed in any animal throughout the administration period. The mean daily food consumption of low‐dose females was significantly lower from Days 57 to 64 and of mid‐dose females was significantly higher from Days 85 to 90, respectively, compared to the control group.

#### 3.2.2 Clinical pathology and urinalysis

No treatment‐related changes in hematology and ophthalmology were observed in any of the dose groups with the exception of a statistically significant decrease in reticulocyte ratio and count in the mid‐dose females compared to the control group (Table 7). A statistically significant increase in γ‐globulin ratio and concentration in high‐dose females was observed compared to the control group (Table 8). No other significant differences were observed in measured blood chemistry parameters. A significant increase was observed in protein‐positive urine in mid‐dose males and high‐dose males and females (data not shown). Similarly, an increase in ketone body‐positive urine in high‐dose animals was observed (data not shown).

**Table 7:** Hematology values for male and female rats administered GT by gavage for 13 weeks

Parameter measured (mean ± *SD*)	Dose group (mg TOS/kg body weight)							
	Males (*n* = 10)				Females (*n* = 10)			
	0 (control)^a^	161	643	2,570	0 (control)^a^	161	643	2,570
Hematocrit (%)	46.1 ± 2.0	46.2 ± 1.3	45.3 ± 1.4	46.4 ± 1.5	44.6 ± 1.9	43.8 ± 1.3	44.2 ± 1.7	43.2 ± 1.4
Hemoglobin (g/dl)	15.5 ± 0.7	15.6 ± 0.5	15.5 ± 0.5	15.6 ± 0.4	15.5 ± 0.5	15.2 ± 0.6	15.2 ± 0.7	15.0 ± 0.5
RBC (×10^6^/mm^3^)	8.64 ± 0.42	8.68 ± 0.18	8.60 ± 0.34	8.61 ± 0.27	8.21 ± 0.35	7.98 ± 0.28	8.14 ± 0.38	7.93 ± 0.26
MCV (µM^3^)	53.3 ± 1.2	53.2 ± 1.2	52.7 ± 1.1	53.9 ± 1.3	54.3 ± 0.9	54.9 ± 1.0	54.3 ± 0.9	54.6 ± 1.0
MCH (pg)	18.0 ± 0.4	18.0 ± 0.5	18.0 ± 0.4	18.1 ± 0.5	18.8 ± 0.3	19.1 ± 0.4	18.7 ± 0.3	19.0 ± 0.4
MCHC (%)	33.8 ± 0.3N	33.8 ± 0.3	34.1 ± 0.2	33.7 ± 0.7	34.7 ± 0.5	34.7 ± 0.5	34.4 ± 0.3	34.8 ± 0.3
Reticulocyte ratio (%)	2.3 ± 0.6N	2.1 ± 0.2	2.3 ± 0.5	2.1 ± 0.3	2.0 ± 0.2	2.2 ± 0.4	1.6 ± 0.3*	2.1 ± 0.4
Reticulocyte count (×10^9^/L)	195.5 ± 41.7N	186.3 ± 15.0	199.6 ± 38.7	178.0 ± 21.7	165.6 ± 18.6	171.6 ± 26.6	131.8 ± 22.0*	165.3 ± 32.3
WBC (×10^3^/mm^3^)	8.27 ± 1.45	8.51 ± 2.29	7.35 ± 1.92	8.91 ± 2.22	4.58 ± 1.10	4.64 ± 1.32	5.18 ± 1.78	4.55 ± 1.59
Differential leukocyte ratios (%)								
Neutrophil (%)	17.4 ± 5.4	19.6 ± 8.0	18.4 ± 4.8	19.3 ± 10.3	15.5 ± 5.1	16.3 ± 5.1	13.0 ± 3.6	13.1 ± 5.9
Lymphocyte (%)	76.7 ± 6.3	74.6 ± 8.2	76.3 ± 4.7	75.8 ± 10.4	78.4 ± 6.0	77.6 ± 5.6	80.6 ± 4.0	81.3 ± 6.4
Monocyte (%)	3.5 ± 1.1	3.3 ± 0.7	2.9 ± 1.0	2.9 ± 0.6	2.6 ± 1.0	3.2 ± 0.9	2.9 ± 0.8	2.6 ± 0.7
Eosinophil (%)	1.5 ± 0.4	1.8 ± 0.6	1.6 ± 0.6	1.3 ± 0.3	2.5 ± 0.8N	2.0 ± 0.4	2.5 ± 0.7	2.1 ± 1.1
Basophil (%)	0.1 ± 0.1	0.1 ± 0.1	0.2 ± 0.1	0.1 ± 0.1	0.1 ± 0.1	0.1 ± 0.0	0.1 ± 0.1	0.1 ± 0.0
LUC (%)	0.8 ± 0.3	0.6 ± 0.2	0.7 ± 0.3	0.7 ± 0.3	1.0 ± 0.3	0.8 ± 0.2	0.9 ± 0.5	0.8 ± 0.3
Differential leukocyte counts (×10^8^/mm^3^)								
Neutrophil (×10^3^/mm^3^)	1.41 ± 0.42N	1.69 ± 0.83	1.30 ± 0.28	1.82 ± 1.50	0.69 ± 0.22	0.76 ± 0.34	0.64 ± 0.19	0.60 ± 0.38
Lymphocyte (×10^3^/mm^3^)	6.38 ± 1.42	6.33 ± 1.81	5.66 ± 1.65	6.64 ± 1.51	3.62 ± 1.04	3.60 ± 1.04	4.20 ± 1.56	3.69 ± 1.32
Monocyte (×10^3^/mm^3^)	0.28 ± 0.07	0.28 ± 0.09	0.22 ± 0.11	0.26 ± 0.11	0.12 ± 0.05	0.15 ± 0.05	0.15 ± 0.06	0.12 ± 0.06
Eosinophil (×10^3^/mm^3^)	0.12 ± 0.04	0.15 ± 0.07	0.11 ± 0.04	0.12 ± 0.04	0.12 ± 0.04	0.09 ± 0.03	0.12 ± 0.02	0.10 ± 0.05
Basophil (×10^3^/mm^3^)	0.01 ± 0.01	0.01 ± 0.01	0.01 ± 0.00	0.01 ± 0.01	0.00 ± 0.01	0.00 ± 0.00	0.01 ± 0.01	0.01 ± 0.01
LUC (×10^3^/mm^3^)	0.07 ± 0.03	0.05 ± 0.02	0.06 ± 0.03	0.07 ± 0.04	0.05 ± 0.02	0.04 ± 0.02	0.05 ± 0.03	0.04 ± 0.02
Blood coagulation analysis								
Platelet (×10^3^/mm^3^)	1,002 ± 187N	1,008 ± 94	1,053 ± 82	1,044 ± 70	1,097 ± 110	1,107 ± 134	1,110 ± 178	1,092 ± 114
PT (seconds)	12.2 ± 1.1	12.2 ± 1.2	12.9 ± 1.6	10.9 ± 1.0	8.9 ± 0.3	8.9 ± 0.2	8.9 ± 0.1	8.8 ± 0.2
APTT (seconds)	23.5 ± 2.0	22.5 ± 2.9	24.7 ± 1.8	21.7 ± 2.4	15.6 ± 2.3	16.4 ± 1.6	16.6 ± 2.1	16.2 ± 1.4

Abbreviations: APTT, activated partial thromboplastin time; GT, glutaminase from nongenetically modified *A. niger* strain GT147; LUC, large unstained cells; MCH, mean corpuscular hemoglobin; MCHC, mean corpuscular hemoglobin concentration; MCV, mean corpuscular volume; *N*, nonparametric analysis; PT, prothrombin time; RBC, red blood cell; *SD*, standard deviation; TOS, total organic solids; WBC, white blood cell.

a Control animals were administered water.

* Significantly different from control (Dunnett's multiple test, *p* ≤ .05).

**Table 8:** Blood chemistry and urinalysis values for male and female rats administered GT by gavage for 13 weeks

Parameter measured (mean ± *SD*)	Dose group (mg TOS/kg body weight)							
	Males (*n* = 10)				Females (*n* = 10)			
	0 (control)^a^	161	643	2,570	0 (control)^a^	161	643	2,570
Blood chemistry								
Glucose (mg/dl)	160 ± 18	162 ± 16	163 ± 15	162 ± 15	155 ± 25	153 ± 17	148 ± 25	152 ± 12
Triglycerides (mg/dl)	61 ± 23	70 ± 18	55 ± 23	72 ± 22	27 ± 10N	35 ± 29	31 ± 12	31 ± 11
Total cholesterol (mg/dl)	61 ± 10	60 ± 14	63 ± 7	55 ± 11	71 ± 12	68 ± 14	66 ± 11	72 ± 10
BUN (mg/dl)	14.3 ± 2.6	13.9 ± 1.7	14.0 ± 2.5	14.8 ± 3.2	15.3 ± 2.8	14.5 ± 2.3	15.8 ± 3.3	14.3 ± 3.1
Creatinine (mg/dl)	0.25 ± 0.05	0.26 ± 0.06	0.26 ± 0.04	0.25 ± 0.05	0.30 ± 0.07	0.30 ± 0.03	0.34 ± 0.08	0.29 ± 0.06
Total bilirubin (mg/dl)	0.06 ± 0.02	0.06 ± 0.01	0.06 ± 0.01	0.05 ± 0.01	0.07 ± 0.02	0.07 ± 0.02	0.08 ± 0.02	0.08 ± 0.03
AST (U/l)	78 ± 29N	73 ± 16	68 ± 7	65 ± 8	69 ± 17N	69 ± 10	80 ± 54	60 ± 9
ALT (U/l)	32 ± 14N	31 ± 7	28 ± 4	29 ± 5	27 ± 11N	23 ± 10	35 ± 46	22 ± 6
ALP (U/l)	306 ± 66N	340 ± 64	314 ± 29	335 ± 107	159 ± 34	154 ± 24	146 ± 36	167 ± 36
*γ*‐GTP (U/l)	0.4 ± 0.2	0.5 ± 0.3	0.3 ± 0.2	0.3 ± 0.2	0.6 ± 0.3	0.5 ± 0.2	0.6 ± 0.3	0.6 ± 0.2
Calcium (mg/dl)	9.52 ± 0.20	9.54 ± 0.19	9.58 ± 0.18	9.57 ± 0.27	6.98 ± 0.34	9.81 ± 0.51	9.75 ± 0.33	9.61 ± 0.27
Inorganic phosphorus (mg/dl)	5.97 ± 0.61	6.04 ± 0.29	5.52 ± 0.39	5.71 ± 0.47	5.06 ± 0.75	4.91 ± 0.69	4.93 ± 0.75	4.74 ± 0.50
Sodium (mmol/L)	143.6 ± 1.4N	144.2 ± 1.3	143.8 ± 0.5	143.9 ± 0.9	142.1 ± 1.4	142.1 ± 0.8	141.4 ± 0.9	141.7 ± 1.3
Potassium (mmol/L)	4.75 ± 0.25	4.65 ± 0.26	4.62 ± 0.17	4.71 ± 0.21	4.39 ± 0.32	4.55 ± 0.30	4.51 ± 0.36	4.48 ± 0.22
Chloride (mmol/L)	107.9 ± 1.4	108.6 ± 1.2	108.3 ± 1.5	108.2 ± 1.4	108.8 ± 0.9	108.8 ± 1.6	108.8 ± 1.5	108.6 ± 1.3
Total protein (g/dl)	5.94 ± 0.22	5.88 ± 0.20	6.08 ± 0.13	5.86 ± 0.23	6.30 ± 0.34	6.49 ± 0.47	6.44 ± 0.33	6.37 ± 0.41
Albumin (g/dl)	2.98 ± 0.17	2.91 ± 0.17	2.99 ± 0.11	2.88 ± 0.12	3.50 ± 0.23	3.64 ± 0.30	3.61 ± 0.31	3.51 ± 0.22
*α*‐1 (g/dl)	1.36 ± 0.16	1.32 ± 0.09	1.38 ± 0.17	1.30 ± 0.21	1.16 ± 0.10N	1.14 ± 0.25	1.16 ± 0.12	1.12 ± 0.14
*α*‐2 (g/dl)	0.40 ± 0.05	0.41 ± 0.07	0.43 ± 0.05	0.43 ± 0.06	0.38 ± 0.05	0.40 ± 0.04	0.38 ± 0.05	0.39 ± 0.05
*β*(g/dl)	0.96 ± 0.08	1.00 ± 0.08	0.99 ± 0.05	0.99 ± 0.05	0.90 ± 0.05	0.96 ± 0.06	0.91 ± 0.07	0.87 ± 0.06
*γ*(g/dl)	0.24 ± 0.08	0.25 ± 0.08	0.28 ± 0.08	0.26 ± 0.06	0.36 ± 0.07	0.35 ± 0.07	0.37 ± 0.10	0.48 ± 0.09*
A/G	1.01 ± 0.10	0.99 ± 0.10	0.97 ± 0.07	0.97 ± 0.10	1.26 ± 0.09	1.29 ± 0.14	1.28 ± 0.13	1.23 ± 0.09
Urinalysis								
Volume (ml)	22.4 ± 6.9	27.0 ± 10.1	23.3 ± 8.3	20.9 ± 5.9	14.5 ± 6.0	14.2 ± 2.9	12.9 ± 5.0	14.9 ± 6.5
Osmotic pressure (mOsm/kg)	896 ± 237	784 ± 278	950 ± 379	1,091 ± 295	1,139 ± 341	1,097 ± 172	1,354 ± 432	1,308 ± 360
Sodium (mmol/L)	38.0 ± 10.9	34.8 ± 22.0	52.4 ± 29.2*	39.5 ± 20.2	69.8 ± 22.1	69.6 ± 13.5	87.4 ± 23.4	80.4 ± 23.3
Potassium (mmol/L)	113.6 ± 32.4	95.7 ± 44.7	121.9 ± 61.5	122.9 ± 41.1	151.7 ± 46.2	151.2 ± 28.2	181.8 ± 57.3	162.7 ± 43.7
Chloride (mmol/L)	57.0 ± 18.8	52.5 ± 31.4	66.6 ± 44.6	67.1 ± 27.3	96.6 ± 30.8	95.0 ± 22.0	120.3 ± 35.3	112.6 ± 32.5

Abbreviations: A/G, albumin/globulin; ALP, alkaline phosphatase; ALT, alanine aminotransferase; AST, aspartate aminotransferase; BUN, blood urea nitrogen; GT, glutaminase from nongenetically modified *A. niger* strain GT147; GTP, glutamyl transpeptidase; *N*, nonparametric analysis; *SD*, standard deviation; TOS, total organic solids.

a Control animals were administered water.

*n* = 9 animals were used for the statistical analysis.

* Significantly different from control (Dunnett's multiple test, *p* ≤ .01).

#### 3.2.3 Pathology

No significant changes in relative and absolute organ weights were observed in any group (Table 9). Upon macroscopic examination, the following findings were observed in various groups: red patches on lungs, nodule on stomach, diverticulum of small intestine, black patches on liver, atrophic kidneys, cyst in kidneys, focal depression on kidneys, soft testes, yellow patches on epididymides, dilated lumen of uterus, and cyst in pituitary gland. Upon histopathological examination, the following findings were observed: hemorrhage in the lungs of 1 male, osseous metaplasia in the lungs of 1 male and 1 female, atrophy of seminiferous tubule in tests, spermatic granuloma in epididymides, and decreased sperm in epididymides of 1 male, and cortical vacuolation in adrenal glands of 1 male at the highest dose. None of the macroscopic or histological changes observed showed any treatment‐related patterns.

**Table 9:** Absolute and relative organ weights of male and female rats administered GT by gavage for 13 weeks

Parameter measured (mean ± *SD*)	Dose group (mg TOS/kg body weight)							
	Males (*n* = 10)				Females (*n* = 10)			
	0 (control)^a^	161	643	2,570	0 (control)^a^	161	643	2,570
Absolute organ weight								
Brain (g)	2.24 ± 0.08	2.21 ± 0.07	2.23 ± 0.09	2.18 ± 0.07	1.98 ± 0.10	2.00 ± 0.08	2.00 ± 0.10	2.03 ± 0.10
Heart (g)	1.46 ± 0.20	1.46 ± 0.12	1.41 ± 0.11	1.55 ± 0.13	0.86 ± 0.08	0.90 ± 0.12	0.89 ± 0.09	0.87 ± 0.05
Liver (g)	12.16 ± 1.84	12.41 ± 1.42	12.64 ± 1.32	13.22 ± 1.49	6.38 ± 0.70	6.55 ± 1.22	6.79 ± 0.52	6.62 ± 0.93
Kidneys (g)	3.10 ± 0.33	3.11 ± 0.22	2.96 ± 0.32	3.29 ± 0.29	1.72 ± 0.14	1.73 ± 0.22	1.86 ± 0.12	1.82 ± 0.15
Spleen (g)	0.69 ± 0.11	0.71 ± 0.08	0.71 ± 0.08	0.78 ± 0.09	0.46 ± 0.05	0.47 ± 0.07	0.48 ± 0.07	0.47 ± 0.09
Adrenal glands (mg)	54 ± 9	55 ± 6	56 ± 14	58 ± 10	59 ± 8	63 ± 9	61 ± 10	57 ± 10
Testes (g)	3.32 ± 0.26	3.31 ± 0.35	3.39 ± 0.22	3.25 ± 0.19	–	–	–	–
Ovaries (mg)	–	–	–	–	70 ± 18	76 ± 12	83 ± 13	77 ± 11
Thymus (mg)	297 ± 46	338 ± 83	285 ± 47	334 ± 74	274 ± 73	249 ± 54	247 ± 38	269 ± 74
Uterus (mg)	–	–	–	–	754 ± 429N	635 ± 179	547 ± 87	705 ± 221
Epididymides (mg)	1,204 ± 84	1,201 ± 70	1,247 ± 111	1,194 ± 147	–	–	–	–
Relative organ weight (%)								
Brain	0.461 ± 0.055	0.444 ± 0.036	0.450 ± 0.046	0.430 ± 0.033	0.741 ± 0.061	0.755 ± 0.088	0.727 ± 0.061	0.751 ± 0.090
Heart	0.296 ± 0.020	0.292 ± 0.013	0.284 ± 0.033	0.305 ± 0.023	0.323 ± 0.031	0.334 ± 0.020	0.322 ± 0.035	0.321 ± 0.033
Liver	2.468 ± 0.165	2.470 ± 0.135	2.533 ± 0.126	2.590 ± 0.226	2.379 ± 0.159	2.424 ± 0.126	2.464 ± 0.142	2.415 ± 0.199
Kidneys	0.633 ± 0.057	0.620 ± 0.027	0.594 ± 0.042	0.645 ± 0.032	0.644 ± 0.061	0.646 ± 0.036	0.678 ± 0.076	0.671 ± 0.069
Spleen	0.141 ± 0.018	0.142 ± 0.018	0.142 ± 0.018	0.153 ± 0.013	0.171 ± 0.024	0.177 ± 0.023	0.176 ± 0.022	0.173 ± 0.030
Adrenal glands	0.011 ± 0.002	0.011 ± 0.002	0.011 ± 0.003	0.011 ± 0.002	0.022 ± 0.003	0.024 ± 0.004	0.022 ± 0.003	0.021 ± 0.003
Testes	0.685 ± 0.111	0.663 ± 0.086	0.691 ± 0.123	0.639 ± 0.049	–	–	–	–
Ovaries	–	–	–	–	0.026 ± 0.007N	0.028 ± 0.003	0.030 ± 0.003	0.028 ± 0.004
Thymus	0.061 ± 0.008	0.067 ± 0.015	0.058 ± 0.011	0.065 ± 0.012	0.103 ± 0.029	0.095 ± 0.027	0.090 ± 0.020	0.098 ± 0.022
Uterus	–	–	–	–	0.283 ± 0.165N	0.241 ± 0.082	0.200 ± 0.041	0.260 ± 0.090
Epididymides	0.249 ± 0.039	0.241 ± 0.020	0.252 ± 0.035	0.235 ± 0.031	–	–	–	–

Abbreviations: GT, glutaminase from nongenetically modified *A. niger* strain GT147; *N*, nonparametric analysis; *SD*, standard deviation; TOS, total organic solids.

a Control animals were administered water.

## 4 DISCUSSION

Sumizyme GT is a liquid concentrate enzyme preparation containing glutaminase from nongenetically modified *A. niger*. Glutaminase has significant commercial use in the production of food ingredients that improve the umami or savory flavor of foods. Traditionally, glutaminase is used in the production of soy sauce by converting l‐glutamine that is formed from the hydrolysis of proteins from soybeans and wheat into l‐glutamic acid, thus imparting a savory flavor (Ohshita et al., 2000). There exists a technological need for glutaminase by the food industry; without glutaminase, l‐glutamine is chemically converted to pyroglutamic acid, which does not have any taste or flavor. The safety of glutaminase derived from nongenetically modified *A. niger* was evaluated in a series of genotoxicity and mutagenicity bioassays, including a bacterial reverse mutation test, mammalian chromosomal aberration test, and in vivo Comet tests, and a 90‐day repeated‐dose oral toxicity study in rats.

While several slight, but statistically significant, positive findings were observed in the studies using the preincubation method (Tables 1 and 2), the enzyme preparation was demonstrated to be nonmutagenic in the bacterial reverse mutation assay at concentrations up to 12.85 mg TOS/plate when evaluated using the treat‐and‐wash procedure. The treat‐and‐wash method is considered to be a valid method to eliminate “false‐positive” findings arising from free amino acids in the test substances, and has been successfully employed in the mutagenicity evaluation of other enzyme preparations and proteinaceous materials using the Ames assay (EFSA, 2014; Okado et al., 2015, 2016, 2019; Thompson et al., 2005). The negative findings using the treat‐and‐wash method demonstrate that any positive or mutagenic effect observed in the studies using the preincubation method may be attributable to the presence of free amino acids, and likewise, the presence of these free amino acids in the test substance likely resulted in the increase in background lawn in the preincubation studies, a finding that was not observed in the treat‐and‐wash studies. Thus, the enzyme preparation was nonmutagenic under the conditions of the Ames test using the treat‐and‐wash method. In the chromosomal aberration assay, the statistically significant concentration‐dependent increases in chromosomal aberrations observed in both the short‐term test (with and without metabolic activation) and in the continuous 24‐hr assay were unlikely to be attributed to a change in pH or osmolality of the test article, as the pH of the medium was comparable between the test concentrations and the negative control, and no precipitation of the test article was observed. Considering that increasing incidence rates of chromosomal aberrations were generally observed at higher concentrations, it is possible that the clastogenic effect was due to disruption of cellular processes, as a result, cytotoxicity, at these high concentrations rather than an inherent toxicological effect of the enzyme (Galloway, 2000). However, significant increases in chromosome aberrations were noted even at concentrations at high relative growth rates (Table 4) rendering the entire cause of the clastogenic action unresolved. Interestingly, similar findings were not reported for glutaminase from *Bacillus amyloliquefaciens* in another chromosomal aberration test with Chinese hamster ovary cells at concentrations up to 200 µg/ml in the presence and absence of S9 metabolic activation (FSANZ, 2015a). Nevertheless, consistent with the requirements of the genotoxicity testing strategy proposed by EFSA, in the event of an inconclusive, contradictory, or equivocal response in an in vitro test, as was observed in the in vitro chromosomal aberration test, the potential genotoxicity of Sumizyme GT was further examined in an in vivo alkaline comet test (EFSA, 2009, 2011). The comet assay was selected as it is considered by EFSA to be a useful indicator to detect gene mutations and/or structural chromosomal aberrations and can be used with many target tissues (EFSA, 2011). Since Sumizyme GT, a proteinaceous/amino acid‐containing material, is not likely to be systemically available due to proteolysis of the enzyme in the stomach and small intestine and would therefore not reach the bone marrow intact (hence a micronucleus test was not performed), the comet tests were performed with stomach and duodenal cells (i.e., target cells due to site of contact). These rapidly dividing cells were examined as they would address any potential site‐of‐contact genotoxicity concerns of the enzyme given the lack of systemic exposure to the enzyme (EFSA, 2011; OECD, 2016a). Adequate and direct exposure of the test article to target tissues/cells is an important consideration in the testing of chemicals for genotoxicity potential, and therefore, the comet tests using stomach and duodenal cells were considered acceptable to further evaluate the genotoxic potential of glutaminase. The significant increase in % tail DNA in the duodenum of rats observed in the initial alkaline comet assay at the highest tested dose (2,570 mg TOS/kg) was within the testing facility's historical control range (0.0 to 4.01%, *n* = 27) and was not reproducible in the second comet assay, and therefore, this finding was deemed to not be related to treatment with Sumizyme GT. Furthermore, no significant changes in hedgehog frequencies in stomach or duodenum cells were observed up to the highest dose that would be suggestive of a cytotoxic effect, and macroscopic examination of both stomach and duodenum did not reveal any toxic effect related to Sumizyme GT administration. Collectively, although the results of the in vitro mammalian chromosomal aberration test produced an unexplained positive effect, the results of the follow‐up in vivo alkaline comet assays in stomach and duodenal cells, as well as the results of the bacterial reverse mutation test, support a lack of genotoxic and mutagenic potential of Sumizyme GT. This conclusion is supported by the stepwise, weight‐of‐evidence approach to genotoxicity testing as described by EFSA (2011).

In the 90‐day repeated‐dose oral toxicity study, the findings related to clinical observations were considered to be spontaneous and not related to Sumizyme GT administration as they were slight, were only observed in a single animal or dose, and were not dose‐dependent.

A slight but significant decrease in mean daily food consumption in low‐dose and an increase in mid‐dose females only was observed in 1 sex. As these effects were reported in 1 sex only and not noted in the higher dose group, as such, they were not considered to be treatment‐related.

Hematological findings were limited to significant decreases in reticulocyte ratio and count in mid‐dose females and increases in γ‐globulin ratio and concentration in high‐dose females. As they were observed only in 1 test group and 1 sex and were not dose‐dependent, these changes were considered to be unrelated to treatment with Sumizyme GT.

The findings from macroscopic and pathological examinations were considered to be spontaneous and incidental, as they were focally distributed, were observed only in a single animal, or were not dose‐dependent. Likewise, no toxicological findings were reported in other subchronic toxicity studies conducted with glutaminase derived from *Cryptococcus albidus* (Ohshita et al., 2000) or *Bacillus amyloliquefaciens* (FSANZ, 2015b). Based on the absence of adverse effects of Sumizyme GT, the NOAEL is considered to be 29,560 U/kg bw per day, equivalent to 2,570 mg TOS/kg bw per day, the highest dose tested. The absence of any histological effects in the gastrointestinal tract and other organs, including the liver, within the 90‐day study at the highest dose tested, as well as the comet assay, further supports the conclusion of a lack of genotoxicity potential.

The safety assessment of a food enzyme must consider the source organism in addition to the inherent hazard profile of the enzyme (Pariza & Johnson, 2001). The safety of *A. niger* for the production of food ingredients and enzymes is well established in the scientific literature and has been extensively reviewed by the U.S. FDA and the Joint FAO/WHO Expert Committee on Food Additives (JECFA) who have collectively raised no safety concerns (JECFA, 1990; US FDA, 2018). In 1989, JECFA established a single acceptable daily intake (ADI) of “not specified” for enzyme preparations of carbohydrases, amyloglucosidases, endo‐1,3(4)‐β‐glucanase, hemicellulose, pectinases, and protease derived from *A. niger*, citing that *A. niger* is a “*common organism in food, that many strains have a long history of use as an enzyme source, and that numerous studies from various preparations from various strains have demonstrated no hazard to human health*” (JECFA, 1990). The production strain used to produce Sumizyme GT was screened for the production of common secondary metabolites and mycotoxins, such as aflatoxin B1, B2, G1, and G2, sterigmatocystin, zearalenone, ochratoxin A, and T‐2 toxin, and was demonstrated analytically to be free of these toxins (personal communication).

The results of the toxicological studies as described herein, together with the history of safe use of the production strain (*A. niger*) in food processing, support the safety of Sumizyme GT for use in food production for human consumption.

## CONFLICT OF INTEREST

The authors of this publication have declared that they have no conflicts of interest.

## ETHICAL APPROVAL

The 90‐day study was conducted in compliance with the Act on Welfare and Management of Animals, and Standards Relating to the Care and Management of Laboratory Animals and Relief of Pain. The study was reviewed and approved by the Institutional Animal Care and use Committee of the Public Interest Incorporated Foundation Biosafety Research Center.

## ENDNOTE

1 All studies were conducted at Biosafety Research Center (Shizuoka, Japan). Biosafety Research Center is a Good Laboratory Practice‐compliant facility. All studies were performed in compliance with the Organisation of Economic Co‐operation and Development (OECD) Principles of Good Laboratory Practice (as revised in 1997) (ENV/MC/CHEM(98)17—OECD, 1998a).
